# Comparison and interpretation of freshwater bacterial structure and interactions with organic to nutrient imbalances in restored wetlands

**DOI:** 10.3389/fmicb.2022.946537

**Published:** 2022-09-21

**Authors:** Fuchao Zheng, Tiange Zhang, Shenglai Yin, Ge Qin, Jun Chen, Jinghua Zhang, Dehua Zhao, Xin Leng, Shuqing An, Lu Xia

**Affiliations:** ^1^School of Life Sciences, Institute of Wetland Ecology, Nanjing University, Nanjing, Jiangsu, China; ^2^Nanjing University Ecology Research Institute of Changshu, Changshu, Jiangsu, China; ^3^College of Life Sciences, Nanjing Normal University, Nanjing, Jiangsu, China

**Keywords:** bacterial assemblages, co-occurrence network, nutrient imbalance, organic matter, restored wetlands

## Abstract

Chemical oxygen demand to nitrogen (COD/N) and nitrogen to phosphorus (N/P) ratios have distinct effects on bacterial community structure and interactions. However, how organic to nutrient imbalances affect the structure of freshwater bacterial assemblages in restored wetlands remains poorly understood. Here, the composition and dominant taxa of bacterial assemblages in four wetlands [low COD/N and high N/P (LH), low COD/N and low N/P (LL), high COD/N and high N/P (HH), and high COD/N and low N/P (HL)] were investigated. A total of 7,709 operational taxonomic units were identified by high throughput sequencing, and Actinobacteria, Proteobacteria, and Cyanobacteria were the most abundant phyla in the restored wetlands. High COD/N significantly increased bacterial diversity and was negatively correlated with N/P (*R*^2^ = 0.128; *p* = 0.039), and the observed richness (Sobs) indices ranged from 860.77 to 1314.66. The corresponding Chao1 and phylogenetic diversity (PD) values ranged from 1533.42 to 2524.56 and 127.95 to 184.63. Bacterial beta diversity was negatively related to COD/N (*R*^2^ = 0.258; *p* < 0.001). The distribution of bacterial assemblages was mostly driven by variations in ammonia nitrogen (NH_4_^+^-N, *p* < 0.01) and electrical conductivity (EC, *p* < 0.01), which collectively explained more than 80% of the variation in bacterial assemblages. However, the dominant taxa Proteobacteria, Firmicutes, Cyanobacteria, Bacteroidetes, Verrucomicrobia, Planctomycetes, Chloroflexi, and Deinococcus-Thermus were obviously affected by variation in COD/N and N/P (*p* < 0.05). The highest node and edge numbers and average degree were observed in the LH group. The co-occurrence networkindicated that LH promoted bacterial network compactness and bacterial interaction consolidation. The relationships between organic to nutrient imbalances and bacterial assemblages may provide a theoretical basis for the empirical management of wetland ecosystems.

## Introduction

Great efforts have long been made to conserve, restore, and construct wetlands because of the important functions and services provided by these ecosystems, including water purification, environmental improvement, and biodiversity maintenance (Rebelo et al., 2018; Sampaio et al., 2018; Chen et al., 2019). Previous studies mainly focused on meeting water index standards based on wetland restoration technology and decreasing the water nutritional index, which is closely related to the interactions between microbes in the aquatic environment (Cardinale, 2011; Brisson et al., 2020; Garibay et al., 2021). The anaerobic bacteria in vertical-flow constructed wetlands usually contribute to organic matter degradation, in turn efficiently decreasing the chemical oxygen demand (COD) (Chang et al., 2015). Proteobacteria play a dominant role in artificial–natural coupled wetlands and significantly affect nitrogen removal due to their metabolic versatility (Zhang et al., 2021). Phosphorus (P)-accumulating organisms take up orthophosphate (PO_4_^3–^) from wastewater under aerobic conditions and then hydrolyze the stored poly-P for survival under anaerobic conditions, which can effectively reduce P pollution (Salehi et al., 2019). However, less attention has been given to the relationship between organic to nutrient imbalances and bacterial assemblages in freshwater environments since water quality standards are met.

In aquatic environments, microbial community patterns are dominantly influenced by several environmental factors and nutritional conditions (Baxter et al., 2012; Wu et al., 2016; Hou et al., 2017) that may themselves be influenced by deterministic processes involving non-random and niche-based mechanisms (Vellend, 2010). For example, environmental filtering by factors such as pH, dissolved oxygen (DO) and salinity greatly impacts the structure of bacterial assemblages (Hou et al., 2017; Shang et al., 2022). Interspecific interaction, *e.g.*, mutualism, competition, and predation, may also shape the patterns of bacterial communities in freshwater lakes (Sadeghi et al., 2021). However, a past study indicated that microbial diversity can be promoted by an increase in nutrition levels under moderate eutrophication (Duarte et al., 2009). Generally, the factors that affect the dynamic variations of microbial communities are the nutritional preferences and metabolic differences of the microorganisms, which are affected by organic matter, nitrogen (N), P and N/P ratios in the environment (Lyu et al., 2017; Aanderud et al., 2018; Chen et al., 2021). Notably, nutrient supply ratios play a vital role in microbial growth and cultivation because specific nutrient balances are needed for microbial growth, and limitation of organic matter, N or P may restrict microbial growth and metabolism (Elser et al., 2007; Jarvie et al., 2018). Lai et al. (2020) found that a high COD/N ratio increased microbial diversity by providing a richer carbon source, and the dominant taxa varied depending on the COD/N ratio. For instance, COD/N ratios of 6 and 12 were beneficial to Actinobacteria, Firmicutes, and Chloroflexi, which mainly participate in the process of denitrification (Lai et al., 2020). Lipizer et al. (2011) revealed that seasonal variations in N/P ratios dramatically affected microbial activities and phytoplankton blooms in the Gulf of Trieste.

Moreover, the interactions of microorganisms, which are greatly affected by the ratios between nutrients, are gradually being highlighted in the literature (Aanderud et al., 2018; Jabir et al., 2020). In some instances, low N/P efficiently decreased the negative interactions among bacterial taxa under P addition, and simultaneously improving C, N, and P fertilizer application decreased bacterial connections, which was attributed to the alleviation of bacterial competition for nitrients (Wei et al., 2020). The bacterial taxa in a lower-C/P environment were more diffuse and less connected due to affluent P (Aanderud et al., 2018). A COD/N ratio greater than 2 was conducive to the cultivation of heterotrophic aerobic bacteria and nitrite-oxidizing bacteria and enhanced the relationship between anammox bacteria and heterotrophic bacteria (Wang et al., 2018). However, little is known about the effects of COD/N and N/P on freshwater bacterial diversity, structure and interactions, especially in freshwater environments with low concentrations of COD, total N (TN), and total P (TP).

In this study, we compared the responses of variation in freshwater bacterial assemblages to different COD/N and N/P ratios in restored wetlands, with the following objectives: (1) to evaluate the effects of COD/N and N/P on bacterial diversity and community differentiation, (2) to analyze the effects of COD/N and N/P on bacterial community composition and dominant taxa, and (3) to depict the effects of COD/N and N/P on bacterial interactions.

## Materials and methods

### Sampling locations

**FIGURE 1:**
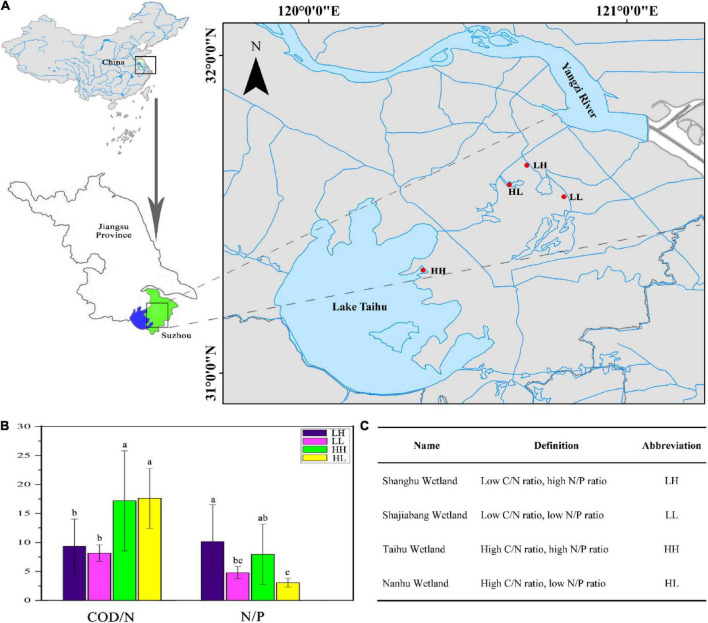
**(A)** Sampling locations in the four wetlands. LH: Shanghu Wetland; LL: Shajiabang Wetland; HH: Taihu Wetland; and HL: Nanhu Wetland. **(B,C)** One-way analysis of variance (ANOVA) of Chemical oxygen demand to nitrogen (COD/N) and nitrogen to phosphorus (N/P) in the four wetlands. Different superscripted lowercase letters indicate *p* < 0.05.

We selected four wetlands that have been restored for more than a decade, and are barely affected by human activities. All the wetlands are located in Lake Taihu Basin, Eastern China. A total of 34 sampling sites were established in the four wetlands in August 2019. Nine sites were sampled each in the Shanghu Wetland (120.6853° E, 31.6537° N), Shajiabang Wetland (120.8021° E, 31.5548° N), and Nanhu Wetland (120.6307° E, 31.5927° N), and seven sites were sampled in the Taihu Wetland (120.3596° E, 31.3238° N) (Figure 1A). Three replicate samples were mixed at each site and a total of 34 freshwater samples (0–10 cm depth) were collected with plastic bottles [polyethylene (PE), volume 2 L, height 22 cm]. We analyzed the COD/N and N/P ratios of freshwater and designated the four wetlands as having low COD/N and high N/P (LH), low COD/N and low N/P (LL), high COD/N and highN/P (HH), or High COD/N and low N/P (HL), with LH, LL, HH, and HL corresponding to the Shanghu, Shajiabang, Taihu and Nanhu wetlands, respectively (Figures 1B,C).

### Environmental parameter measurements

The physical parameters of the surface freshwater were measured *in situ* using a multiprobe instrument (HQd Portable Meter, Edition 6, HACH, USA) before sample collection. The measured parameters included pH, electrical conductivity (EC), and DO. Moreover, TN, TP, COD, nitrate nitrogen (NO_3_^–^-N) and ammonia nitrogen (NH_4_^+^-N) were measured by a water quality analysis system (DRB200 and DR3900, HACH, USA). The biochemical oxygen demand (BOD_5_) and chlorophyll-a (Chla) concentration were determined with the HJ505-2009 and HJ897-2017 methods (Ministry of Ecology and Environment of the People’s Republic of China, 2009, 2017), respectively. The freshwater samples were passed through microporous membranes with a pore size of 0.22°μm and a diameter of 50 mm (Millipore, USA), and microbiological analysis was then conducted on microorganisms attached to the membranes. The microbial filter membrane samples were stored at −80°C until DNA extraction.

### Microorganism measurement and analysis

Microbial community genomic DNA was extracted from freshwater samples using the FastDNA^®^ SPIN Kit (Omega Biotek, Norcross, GA, USA) following the manufacturer’s instructions. The DNA extract was checked on a 1.0% agarose gel, and DNA concentration and purity were determined with a NanoDrop 2000 UV–vis spectrophotometer (Thermo Scientific, Wilmington, DE, USA). The hypervariable V3–V4 region of the bacterial 16S rDNA gene was amplified with the primer pair 338F (5′-ACTCCTACGGGAGGCAGCAG-3′) and 806R (5′-GGACTACHVGGGTWTCTAAT-3′) (Caporaso et al., 2011) by an ABI GeneAmp^®^ 9700 PCR thermocycler (ABI, CA, USA). PCR amplification of the 16S rDNA gene was performed as follows: initial denaturation at 95°C for 3 min; followed by 27 cycles of denaturing at 95°C for 30 s, annealing at 55°C for 30 s and extension at 72°C for 45 s; a single extension at 72°C for 10 min; and a final extension at 4°C. The PCR mixtures contained 5 × TransStart FastPfu buffer 4°μL, 2.5 mM dNTPs 2°μL, forward primer (5°μM) 0.8°μL, reverse primer (5°μM) 0.8°μL, TransStart FastPfu DNA Polymerase 0.4°μL, template DNA 10°ng, and enough ddH_2_O to reach a total volume of 20°μL. PCRs were performed in triplicate. PCR products were extracted from a 2.0% agarose gel, purified using the AxyPrep DNA Gel Extraction Kit (Axygen Biosciences, Union City, CA, USA) and quantified using a Quantus Fluorometer (Promega, USA). The library was constructed using the NEXTFLEX Rapid DNA-Seq Kit, the Illumina MiSeq PE300 platform was used for sequencing, and bacterial DNA fragments of freshwater samples were obtained. The raw 16S rDNA gene sequencing reads were demultiplexed, quality-filtered using Trimmomatic and merged with FLASH 1.2.11 (Center for Computational Biology, Baltimore, MD, USA) (Magoc and Salzberg, 2011). Operational taxonomic units (OTUs) were clustered with a 97% similarity cutoff using UPARSE v.7.1 (Edgar, 2013), and chimeric sequences were identified and removed. The taxonomy of each representative OTU sequence was assigned by RDP Classifier v.7.1 (Wang et al., 2007) against the 16S rRNA database v.132 by using a confidence threshold of 0.7 (Quast et al., 2013).

### Data analysis

One-way analysis of variance (ANOVA) was used to test for differences in the environmental parameters between groups. We used the observed richness (Sobs), Shannon, Chao1, and whole-tree phylogenetic diversity (PD) indices to analyze bacterial diversity, which were calculated with QIIME (Version 1.7.0, Mothur v.1.30.2) (Schloss et al., 2009) and tested for differences with ANOVA. Linear regression analysis was performed to test for relationships of alpha diversity with COD/N and N/P. Pearson correlation analysis was performed between the environmental parameters and diversity indices. The Bray–Curtis distance matrices of bacterial genera were visualized using unconstrained principal coordinate analysis (PCoA), and the correlations of environmental parameters were analyzed with redundancy analysis (RDA) in CANOCO 5.0 (Wageningen University and Research, Wagenin–gen, Netherlands). Rank regression analysis based on PCoA was used to test for correlations between environmental factors and beta diversity (Ren et al., 2022). Permutational multivariate ANOVA (Adonis test) based on Bray–Curtis distances was performed between the groups (Hartman et al., 2018). Community composition analysis was carried out with the “*vegan*” package (R Studio Inc., Massachusetts, USA). Random forest (RF) analysis was used to identify the important bacterial taxa that responded to organic and nutrient ratios (Ren et al., 2022). We identified the 10 most abundant phyla and classes in the bacterial assemblages which were analyzed by ANOVA. Duncan’s test was performed to determine the statistical significance of differences (*p* < 0.05) in bacterial abundance among the dominant in the four groups. Pearson correlation analysis was used to test for correlations between environmental parameters and the 10 most abundant phyla and classes. All statistical tests were performed with the statistical program SPSS 25.0 (SPSS Inc., Chicago, IL, USA).

To analyze the effects of freshwater COD/N and N/P on the bacterial interactions, we calculated the spearman correlation coefficients between bacterial genera using the corr.test function and “*psych*” (Brisson et al., 2019). All bacterial sequencing data analyses were performed in R v3.6.1. Spearman correlations with a magnitude *r* > 0.6 or *r* < −0.6 and statistically significant at *p* < 0.05 were included. To analyze the relationships of highly abundant taxa, we adjusted the filter threshold to identify abundant taxa with an overall frequency greater than 0.5% among all the samples and used them to construct a bacterial co-occurrence network (Chen and Wen, 2021). A set of network topological properties (e.g., nodes, edges, degree, diameter, density modularity coefficient, and average cluster coefficient) were calculated. Bacterial genera with the highest standardized scores for high degree, high closeness centrality, high transitivity, and low betweenness centrality were statistically identified as the keystone taxa (Berry and Widder, 2014). Networks were visualized using the interactive platform Gephi (Bastian et al., 2009).

## Results

### Alpha diversity

The results showed that 90% of the physicochemical parameters significantly differed among the groups (*p* < 0.05, Table 1). For example, the COD concentrations ranged from 8.33 to 22.14 mg/L, and the highest value appeared in the HH group. The NH_4_^+^-N and NO_3_^–^-N concentrations ranged from 0.015 to 0.187 mg/L and 0.385 to 0.600 mg/L, respectively, and both were highest in the LL group. The values of TP ranged from 0.138 to 0.348 mg/L, and the highest value appeared in the HL group (Table 1).

**TABLE 1:** Physicochemical properties (mean ± SE, *n* = 34) of freshwater in the four wetlands in Jiangsu, China.

	pH	EC (μ s⋅cm^–1^)	DO (mg⋅L^–1^)	COD (mg⋅L^–1^)	NH_4_^+^-N (mg⋅L^–1^)
LH	9.274 ± 0.169^a^	349.888 ± 9.943^b^	10.027 ± 0.635^a^	8.333 ± 1.054^c^	0.042 ± 0.008^b^
LL	8.384 ± 0.084^c^	415.555 ± 5.527^a^	4.464 ± 0.529^b^	9.444 ± 1.094^c^	0.187 ± 0.045^a^
HH	9.071 ± 0.142^ab^	325.571 ± 3.637^c^	9.620 ± 0.422^a^	22.142 ± 3.269^a^	0.160 ± 0.024^a^
HL	8.738 ± 0.022^b^	359.000 ± 3.184^b^	5.777 ± 0.481^b^	16.444 ± 1.434^b^	0.015 ± 0.006^b^
	**NO_3_^–^-N (mg⋅L^–1^)**	**TN (mg⋅L^–1^)**	**TP (mg⋅L^–1^)**	**BOD_5_ (mg⋅L^–1^)**	**Chla (μg⋅L^–1^)**
LH	0.422 ± 0.027^b^	1.066 ± 0.166^a^	0.138 ± 0.025^b^	5.222 ± 0.296^c^	4.519 ± 0.758^c^
LL	0.600 ± 0.076^a^	1.166 ± 0.132^a^	0.243 ± 0.017^ab^	7.488 ± 0.312^b^	5.919 ± 0.445^c^
HH	0.385 ± 0.014^b^	1.585 ± 0.478^a^	0.235 ± 0.054^ab^	9.242 ± 0.470^a^	14.493 ± 2.997^b^
HL	0.477 ± 0.052^ab^	0.966 ± 0.072^a^	0.348 ± 0.054^a^	4.355 ± 0.232^c^	20.559 ± 1.739^a^

Different lowercase letters indicate significant differences (*p* < 0.05) among the different sampling areas; LH: Shanghu Wetland; LL: ShajiabangWetland; HH: Taihu Wetland; and HL: Nanhu Wetland. The same applies below.

A total of 1,719,396 16S rDNA sequences were selected for classification from freshwater, and 7,709 OTUs were obtained. The most common sequence length was approximately 414 bp. The alpha diversity indices showed significant differences among groups (*p* < 0.05, Table 2). Specifically, the Shannon indices ranged from 4.16 to 4.55, and that of the LL group was the lowest. The Sobs, Chao1, and PD values ranged from 860.77 to 1314.66, 1533.42 to 2524.56, and 127.95 to 184.63, respectively, and were markedly higher in the HL group than in the LL group (*p* < 0.01, Table 2).

**TABLE 2:** Observed bacterial community richness and diversity indices (mean ± SE, *n* = 34) for the freshwater of four wetlands.

	Sobs	Shannon	Chao1	PD
LH	1207.44 ± 171.13^ab^	4.39 ± 0.18^a^	2077.60 ± 301.22^ab^	169.05 ± 20.00^ab^
LL	860.77 ± 45.76^b^	4.16 ± 0.11^a^	1533.42 ± 126.75^b^	127.95 ± 5.99^b^
HH	1099.71 ± 63.23^ab^	4.55 ± 0.21^a^	2016.19 ± 137.15^ab^	167.45 ± 8.32^ab^
HL	1314.66 ± 111.60^a^	4.50 ± 0.11^a^	2524.56 ± 243.52^a^	184.63 ± 13.13^a^

Different lowercase letters indicate significant differences (*p* < 0.05) among the different sampling areas.

**FIGURE 2:**
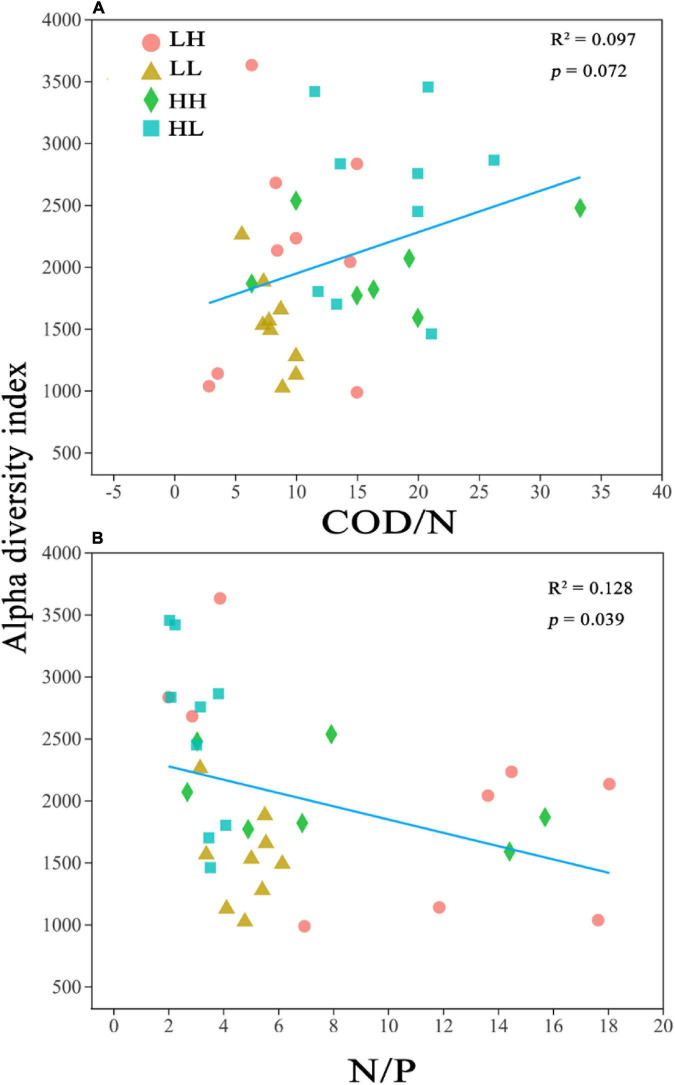
Relationship between bacterial alpha diversity (Chao1) and **(A)** Chemical oxygen demand to nitrogen (COD/N) and **(B)** nitrogen to phosphorus (N/P). *R*^2^ represents the correlation coefficient, *p* < 0.05 indicates significance and *p* > 0.05 indicates non-significance.

The alpha diversity was closely correlated with COD/N (*R*^2^ = 0.097; *p* = 0.072) and N/P (*R*^2^ = 0.128; *p* = 0.039) (Figure 2). Pearson’s correlation analysis performed on the alpha diversity indices and several physicochemical parameters revealed that TP was significantly correlated with Sobs (*r* = 0.352; *p* < 0.05), Chao1 (*r* = 0.501; *p* < 0.01), and PD (*r* = 0.384; *p* < 0.05), and the N/P ratio was significantly correlated with the Sobs (*r* = −0.342; *p* < 0.05), Chao1 (*r* = −0.358; *p* < 0.05), and PD indices (*r* = −0.342; *p* < 0.05) (Appendix Supplementary Table 1).

### Beta diversity and environmental effects

**FIGURE 3:**
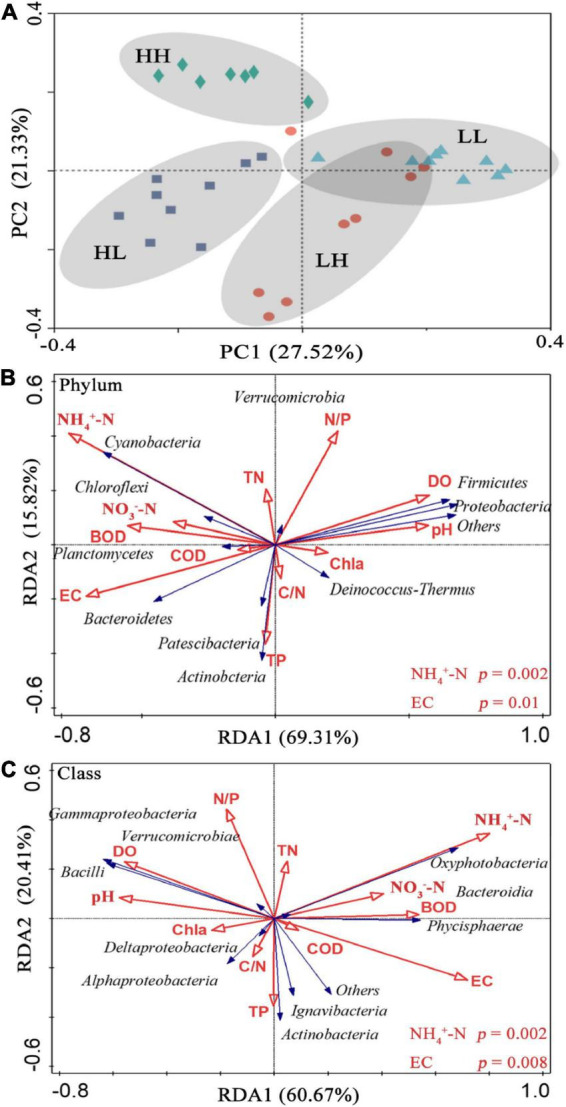
**(A)** Principal coordinate analysis (PCoA) of bacteria based on Bray–Curtis dissimilarity in freshwater of the four wetlands; redundancy analysis (RDA) diagram illustrating the relationships between the compositions of freshwater bacteria at the **(B)** phylum, and **(C)** class levels from different sampling sites with variable environments. Blue arrows show bacterial community composition; red arrows show physicochemical properties in the freshwater.

**FIGURE 4:**
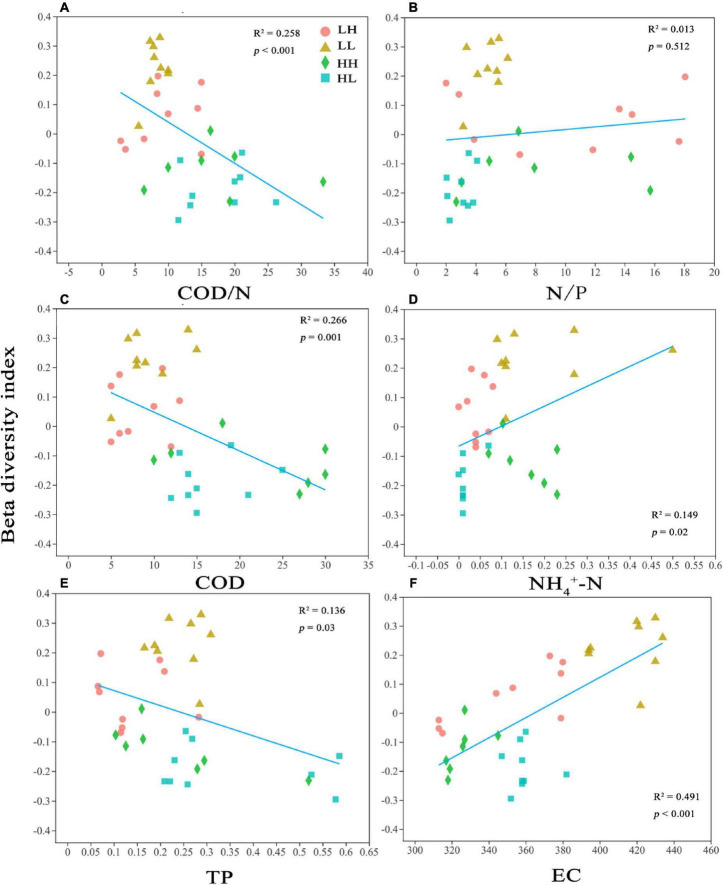
Relationships between bacterial beta diversity and **(A)** Chemical oxygen demand to nitrogen (COD/N), **(B)** nitrogen to phosphorus (N/P), **(C)** Chemical oxygen demand (COD), **(D)** ammonia nitrogen (NH_4_^+^-N), **(E)** total P (TP), and **(F)** electrical conductivity (EC). *R*^2^ represents the correlation coefficient; *p* < 0.05 indicates significance and *p* > 0.05 indicates non-significance.

In the PCoA, PC 1, and PC 2 explained 27. 52 and 21.33% of the variation, respectively, and the two axes together explained 48.85%. The two axes divided the 34 sample locations into four clusters. The LH and LL groups were separated from the HH and HL groups, and the three locations of the LH group overlapped with those of the LL group (Figure 3A). Thus, the bacterial assemblages in the LH and LL groups differed from those in the HH and HL groups. The Adonis test showed that the bacterial assemblages were significantly different between all pairs of groups (Supplementary Table 2; *p* < 0.01). The RDA1 and RDA2 axes explained 69.31 and 15.82%, respectively, of the variation in the physicochemical parameters, and in the RDA of dominant bacterial phyla and classes, they explained 60.67 and 20.41%, respectively, collectively explaining more than 81.08% of the variation in bacterial assemblages. In addition, NH_4_^+^-N and EC had a significant influence on the distribution of bacterial phyla and classes, especially some typical bacterial taxa (Figures 3B,C). In addition, we analyzed the relationships between environmental factors and beta diversity, and the results indicated that COD and COD/N were negatively and significantly related to beta diversity (*R*^2^ = 0.435 and 0.332, respectively; *p* < 0.001), while NH_4_^+^-N was positively connected with beta diversity (*R*^2^ = 0.149 and *p* = 0.02) (Figures 4A,C,D). There was not a significant correlation between N/P and beta diversity (*R*^2^ = 0.016; *p* = 0.467), and TP presented a negative and significant relationship with beta diversity (*R*^2^ = 0.136 and *p* = 0.02) (Figures 4B,E). Moreover, a positive and significant relationship was observed between EC and beta diversity (*R*^2^ = 0.491; *p* < 0.001) (Figure 4F).

### Taxonomy of and variation in bacterial assemblages

**FIGURE 5:**
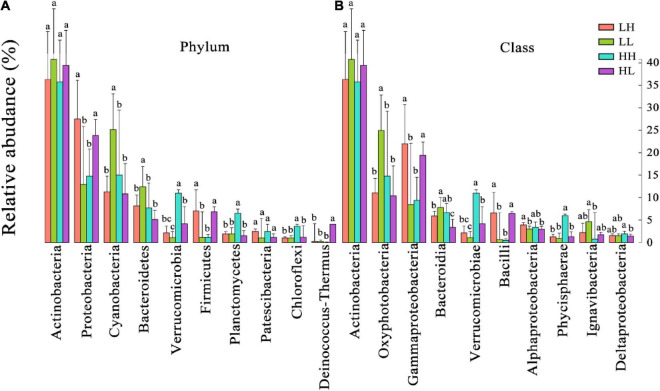
The relative abundances of the 10 most abundant bacteria at the **(A)** phylum and **(B)** class levels in freshwater of the four wetlands. Different superscripted lowercase letters indicate *p* < 0.05.

**FIGURE 6:**
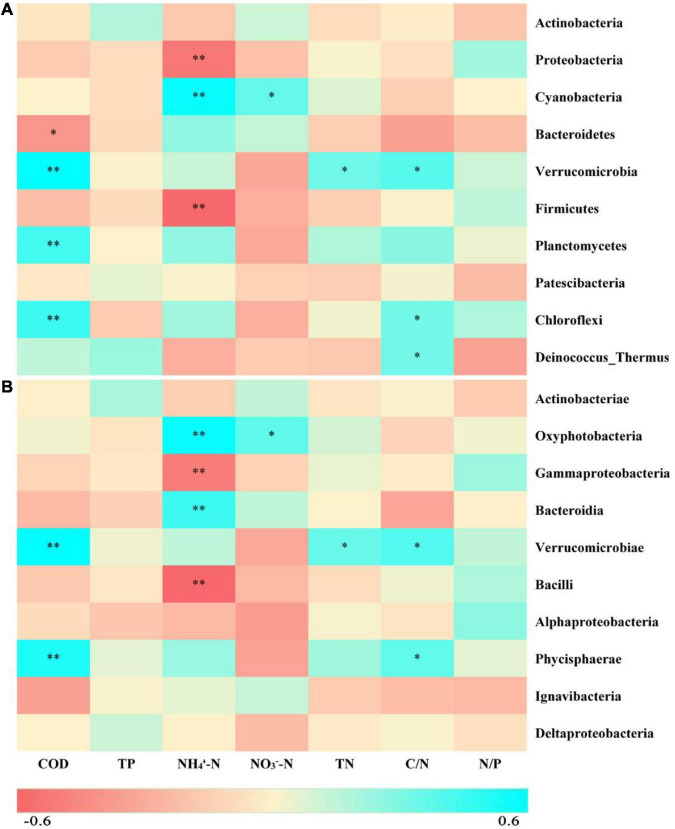
Heatmap depicting correlations between physicochemical properties of the freshwater and the 10 most abundant bacteria at the **(A)** phylum and **(B)** class levels. Red indicates negative correlations, while cyan indicates positive correlations. ** represents *p* < 0.01, and * represents *p* < 0.05.

According to the taxonomic identification results, Actinobacteria, Proteobacteria, and Cyanobacteria were the most abundant phyla, and Oxyphotobacteria, Gammaproteobacteria, and Bacteroidia were the most abundant classes, as shown in Supplementary Figure 1. RF analysis indicated that Planctomycetes, Cyanobacteria, Actinobacteria, Bacteroidetes, and Proteobacteria were the most important phyla and that Planctomycetacia, Oxyphotobacteria, Actinobacteria, Bacteroidia, Gammaproteobacteria, and Alphaproteobacteria were the key bacterial classes that responded to the variation in COD/N and N/P (Supplementary Table 3). The proportions of both the dominant bacterial phyla and classes in freshwater varied among the groups. At the phylum level, the LH and HL groups showed significantly increased abundances of Proteobacteria and Firmicutes (*p* < 0.01), and the LL group showed large increases in the abundance of Cyanobacteria and Bacteroidetes (*p* < 0.01). The abundance of Verrucomicrobia, Planctomycetes, and Chloroflexi significantly increased in the HH group (*p* < 0.01), and the abundance of Deinococcus-Thermus significantly increased in the HL group (*p* < 0.01) (Figure 5A). At the class level, a markedly increased abundance of Oxyphotobacteria was observed in the LL group (*p* < 0.01), significantly improved richness of Gammaproteobacteria and Bacilli was also found in both the LH and HL groups (*p* < 0.01), and the HH group had significantly increased abundances of Verrucomicrobiae and Phycisphaerae (*p* < 0.01). However, the richness of Bacteroidia was decreased in the HL group (*p* < 0.01) (Figure 5B). According to the correlation analysis of the main physicochemical parameters and the 10 most abundant phyla and classes, Verrucomicrobia (*r* = 0.636; *p* < 0.01), Planctomycetes (*r* = 0.469; *p* < 0.01), Chloroflexi (*r* = 0.487; *p* < 0.01), Phycisphaerae (*r* = 0.556; *p* < 0.01), and Bacteroidetes (*r* = −0.345; *p* < 0.05) were significantly correlated with COD. In addition, the NH_4_^+^-N concentration was significantly associated with Cyanobacteria (*r* = 0.619; *p* < 0.01), Oxyphotobacteria (*r* = 0.623; *p* < 0.01), Bacteroidia (*r* = 0.479; *p* < 0.01) and Proteobacteria (*r* = −0.461; *p* < 0.01), and the NO_3_^–^-N concentration was significantly related to Cyanobacteria (*r* = 0.384; *p* < 0.05) and Oxyphotobacteria (*r* = 0.386; *p* < 0.05). Furthermore, TN and COD/N were significantly correlated with Verrucomicrobiae (*r* = 0.359 and 0.415; *p* < 0.05), while COD/N was also related to Chloroflexi (*r* = 0.343; *p* < 0.05), Deinococcus-Thermus (*r* = 0.342; *p* < 0.05), and Phycisphaerae (*r* = 0.381; *p* < 0.05) (Figure 6).

### Variation in the bacterial co-occurrence network

**FIGURE 7:**
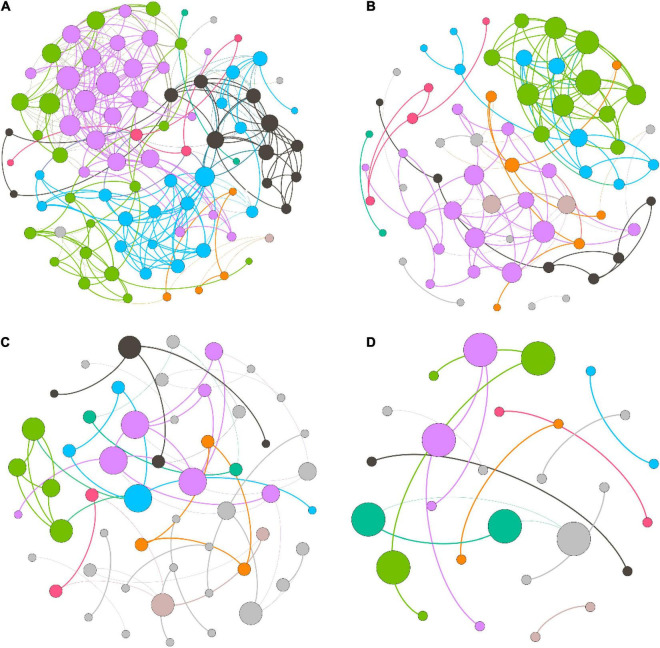
Co-occurrence network of freshwater bacterial assemblages in different wetlands. **(A)** high N/P (LH), **(B)** low N/P (LL), **(C)** high N/P (HH), and **(D)** low N/P (HL). Each node represents a bacterial genus, the size of the node represents the degree, and nodes of the same color represent a network module. The connections between nodes represent significant relationships between genera (*r* > 0.6 or *r* < –0.6, *p* < 0.05), and the thickness of the lines connecting nodes represent the size of the correlation coefficient.

We measured the topological properties of the obtained networks to examine differences in bacterial taxon correlations among the groups (Figure 7). The largest numbers of nodes and edges were observed in the LH group, whereas the lowest numbers appeared in the HL group. The average degree was 7.816, 4.061, 2.151, and 1.259 in the LH, LL, HH, and HL groups, respectively. Similarly, for the diameter of the networks, the highest value also appeared in the LH group, and the lowest value was observed in the HL group. Additionally, the peak network density was detected in the LH group, whereas the lowest value appeared in the HH group, and the highest modularity coefficient and average cluster coefficient were observed in the HH group, while the lowest values appeared in the LL and HL groups (Table 3). However, the keystone taxa obviously differed among these groups. For example, the predominant taxa of the LH group were *Paludibaculum*, WWE3, *Gemmatimonas*, Actinobacteria, Gemmataceae, *Silvanigrella*, Sporichthyaceae, and *Chryseomicrobium*; the major taxa of the LL group were *Mycobacterium*, *Clostridium_sensu_stricto_1*, Pedosphaeraceae, *Roseomonas*, *Aurantimicrobium*, *29_marine_group* and Mitochondria; Rhodocyclaceae, PeM15, *Noviherbaspirillum*, Chloroplast, and *Terrimonas* were mainly present in the HH group; and Sphingomonadaceae, *Chryseobacterium*, Gammaproteobacteria, Subgroup_6, *Pseudorhodobacter*, *Rhodobacter*, Limnobacter, Brevundimonas, and OPB56 were dominant in the HL group, as shown in the Supplementary Tables.

**TABLE 3:** Network topological properties of freshwater bacterial communities in the four wetlands.

	Nodes^a^	Edges^b^	Average degree^c^	Diameter^d^	Density^e^	Modularity coefficient^f^	Average clustering coefficient^g^
LH	87	340	7.816	10	0.091	1.226	0.662
LL	66	134	4.061	9	0.062	0.795	0.548
HH	53	57	2.151	5	0.041	1.584	0.785
HL	17	27	1.259	3	0.048	1.051	0.429
Total	234	2804	23.966	8	0.103	1.637	0.505

^a^Number of species with at least one correlation > 0.6 or < −0.6, and statistically significant at *p* < 0.05.

^b^Number of strong and significant correlations between nodes.

^c^Node connectivity depicts how many connections (on average) each node has to another unique node in the network.

^d^The longest distance between the nodes in the network.

^e^The number of edges divided by the number of edges of a complete graph with the same number of vertices.

^f^A value > 0.4 indicates that the partition produced by the modularity algorithm can be used to detect distinct communities within the network. This indicates that there are nodes in the network that are more densely connected to each other than with the rest of the network and that their density is noticeably higher than the graph’s average density.

^g^How nodes are embedded in their neighborhood and thus the degree to which they tend to cluster together.

## Discussion

Bacterial assemblages are enriched when nutrients or organic matter is abundant, and COD/N is a crucial determinant of the bacterial diversity in freshwater (Han et al., 2016; Lai et al., 2020). With increasing nutrient levels, bacterial diversity can increase under moderate eutrophication and decrease under hypereutrophication (Duarte et al., 2009; Zhang et al., 2022). In this study, we found that a high COD/N (HH group) significantly increased bacterial diversity (Table 2). A negative and significant relationship was observed between N/P and alpha diversity (Figure 2B), and TP presented a positive and significant correlation with Sobs, Chao1 and PD indices (Supplementary Table 1), indicating that a higher N/P ratio of freshwater may have a negative effect on bacterial diversity. A higher N/P ratio may result in P limitation, which is not conducive to the growth of bacterial communities (Cui et al., 2018; Jarvie et al., 2018). Among the N sources, NO_3_^–^-N showed the lowest concentration in the HH group, whereas the concentrations of both NH_4_^+^-N and TN were lowest in the HL group (Table 1). Consistent with our study, several previous studies also revealed that autotrophic nitrifying bacteria, Betaproteobacteria, and Gammaproteobacteria, contributed to N depletion, which promoted a high COD/N (Begum and Batista, 2013; Wang et al., 2019). In terms of nutritional balance, bacterial diversity usually increases when the COD/N increases under conditions of both sufficient N and hypereutrophication (Duarte et al., 2009; Jarvie et al., 2018). However, there were no significant relationships between COD or TN and bacterial diversity (Supplementary Table 1). Thus, the results confirmed that nutritional balance is more important than a single nutrient for the growth of bacterial assemblages, and a higher COD/N increases the diversity of bacterial assemblages in freshwater environments.

Our results revealed that variation in COD/N and N/P obviously impacted the structure of bacterial assemblages in the freshwater environments (Figure 3A), which was consistent with the findings of a previous report (Jabir et al., 2020). Although organic matter is usually regarded as an energy substance, N and P sources are frequently deficient in freshwater environments, which further restrict microbial growth (Elser et al., 2007; Jarvie et al., 2018). Thus, fluctuations in N and P are considered the major factors separating bacterial assemblages (Figures 4D,E). However, NH_4_^+^-N may be one of the most important factors regulating the distribution of bacterial assemblages (Figures 3B,C), which is consistent with the results of several previous studies (Baxter et al., 2012; Wu et al., 2016). First, the bacterial taxa prefer to use NH_4_^+^-N as the electron donor for energy metabolism, which can significantly affect bacterial assemblage structure (Li et al., 2020). Second, NH_4_^+^-N can serve as the N source for ammonia-oxidizing bacteria or archaea through its oxidation to hydroxylamine and conversion to nitrite or nitrate due to diverse pathways derived from exogenous inputs or litter decomposition (Gusewell and Gessner, 2009; Kuypers et al., 2018; Yang et al., 2020). The results indicated that COD/N was negatively related to beta diversity (Figure 4A) and suggested that a higher COD/N ratio was favorable to bacterial differentiation mainly due to COD effects. The main reasons may be that higher organic matter availability facilitates bacterial reproduction, and bacteria with rapid propagation may lead to community differentiation (Blazˇina et al., 2009).

In addition, our results revealed that EC was a key factor explaining variation in the bacterial assemblages (Figures 3B,C), and a significant linear relationship was observed between EC and beta diversity (Figure 2F). These results indicated that EC played a major role in dividing bacterial communities. Korber et al. (1996) reported that high EC negatively influenced bacterial diversity, indicating the bactericidal effect of salinity. EC is related to salinity, which is signified by more extensive anions and cations in the freshwater environment and can limit ion uptake by microorganisms due to osmotic potential and ion competition (Mavi and Marschner, 2013; Ma et al., 2016). A past study showed that EC can potentially impact nutrient bioavailability by mediating alterations in bacterial assemblages (Hessini et al., 2019). Thus, our results showed that beta diversity increased with increasing EC (Figure 4F), indicating that the bacterial community is more stable in freshwater and that EC is a mechanism of positive selection on bacteria. In summary, the differences in bacterial assemblages were driven by organic to nutrient imbalances, COD/N, especially COD, presented negative selection on bacteria, and NH_4_^+^-N and EC also synergistically influenced the distributions of bacterial assemblages.

The structure and relative abundances of bacterial phyla and classes identified in the freshwater samples of different groups were revealed in this study (Figure 5 and Supplementary Figure 1). In general, Actinobacteria are dominant in soil and aquatic surroundings (Newton et al., 2011; Han et al., 2016). Similarly, this study also showed that Actinobacteria were the most abundant bacterial group (Figure 5 and Supplementary Figure 1). Meanwhile, similar abundances of Actinobacteria among the groups were found in this study, which might be attributable to their extensive nutritional adaptability under eutrophy or oligotrophy (Yan et al., 2021). In addition, our results indicated that variations in COD/N and N/P significantly influenced the 10 most abundant bacterial taxa at both the phylum and class levels (Figure 5). For example, Gammaproteobacteria (Proteobacteria), which make up a large number of heterotrophic and mixotrophic species (Gong et al., 2016), and Bacilli (Firmicutes), which are chemoorganotrophs and mainly related to carbon sources (Gaugué et al., 2013), were both closely related to NH_4_^+^-N and affected by the imbalance of nutrients (*e.g.*, in the LH and HL groups) (Figures 5, 6). Although Bacteroidetes and Oxyphotobacteria were also significantly correlated with NH_4_^+^-N, they increased in the LL group because of the low organic matter (Figures 5, 6). Bacteroidetes are heterotrophic taxa and play a key role in polysaccharide degradation (Unfried et al., 2018). Oxyphotobacteria (Cyanobacteria) are photoautotrophs and are sensitive to fluctuations in nitrogen sources (Lindell et al., 2002; Chen and Bibby, 2005), and it has been shown that the reproductive rate of Cyanobacteria is affected by alterations in the concentrations of N and P (Gobler et al., 2016; Paerl, 2017). Moreover, Verrucomicrobia, Chloroflexi, and Phycisphaerae were significantly related to COD and were improved by high organic matter and N concentrations (*e.g.*, in the HH group, Figures 5, 6). As previously reported, Verrucomicrobia is heterotrophic and able to degrade organic matter (Wang et al., 2019), and Chloroflexi can scavenge organic compounds derived from an anammox reactor (Kindaichi et al., 2012). Phycisphaerae-related (Planctomycetes) microbial ecological processes include anammox and mineral encrustation (Shu et al., 2011). Notably, Deinococcus-Thermus can utilize organic matter produced by autotrophs (Kadnikov et al., 2021) and are promoted by rich organic matter and P sources (*e.g.*, in the HL group, shown in Figure 5A). Nonetheless, significant decreases in the abundance of Bacteroidia (Bacteroidetes) were found in the HL group (Figure 5B), possibly due to Bacteroidia being chemo-organoheterotrophs with growth on carbohydrates or peptide mixtures and proteins (Podosokorskaya et al., 2020). Therefore, the dominant taxa presented different ecotypes, nutritional preferences, and responses to nutrient fluctuations.

Previous studies have shown that bacterial assemblages have the ability to exploit resources efficiently, which manifests as a more complex co-occurrence network, when the relationships between bacterial taxa are stronger (Aanderud et al., 2018; Santia et al., 2019). In this study, the co-occurrence network revealed that the bacterial assemblages in the low-COD/N groups presented many more nodes and connections than those in the high-COD/N groups, and the high-N/P groups showed improved network complexity under the same COD/N conditions (Figure 7). Our findings were consistent with those of several previous studies (Aanderud et al., 2018; Jabir et al., 2020). Bacterial taxa form several microbiotas (modules) based on their connections and interactions, which may be due to similar nutritional preferences or resource complementarity (Zheng et al., 2021). Furthermore, more nodes and connections appeared in the low-COD/N and high-N/P groups (Figures 7A,B). One possible explanation for this result is that the bacterial taxa needed a nutrient source with greater reuse efficiency, and the higher N concentrations helped increase the connections between interacting bacteria (Jarvie et al., 2018). While higher bacterial diversity appeared under high COD/N, fewer taxa and connections were observed (Figures 7C,D). One possible explanation is that sufficient organic matter may lead to less nutrient exchange as a result of less nutritional competition among bacterial taxa (Gralka et al., 2020). However, the keystone taxa sensitive to nutritional imbalances were identified in the co-occurrence networks, which greatly differed among the groups (Supplementary Tables). Previous studies have indicated that keystone taxa play a vital role in pollutant degradation, resource transformation, and ecosystem stability maintenance (Banerjee et al., 2018; Shi et al., 2022; Zhang et al., 2022). For instance, the keystone taxa of Sporichthyaceae (Actinobacteria) under high N (*e.g.*, in the LH group) can use various carbohydrates and nitrite (Tamura, 2014), which explains their importance in maintaining the efficient utilization of carbon sources by the microbiota. *Mycobacterium* (Actinobacteria) has strong acid resistance and the ability to grow on simple substrates (*e.g.*, in the LL group) (Nakai et al., 2015), and this taxon may contribute to bacterial assemblage stability under acid perturbation. Members of Rhodocyclaceae (Proteobacteria) prefer a high-organic matter and high-N source environment (*e.g.*, in the HH group) and can play a crucial role in water purification processes, such as carbon source provision, ammonium degradation and nitrogen fixation (Oren, 2014; Aanderud et al., 2018). Sphingomonadaceae (Proteobacteria) dominate in high-organic matter environments (*e.g.*, in the HL group) and perform many functions related to metabolizing and degrading aromatic and heterocyclic compounds, as well as producing extracellular biological polymers, which have shown strong adaptability to surrounding humic surface water and oxidative stress (Glaeser and Kämpfer, 2014). Thus, the fluctuation of nutrient imbalances, especially in relation to N sources, was the determining factor of bacterial network complexity and impacted the diverse functions of keystone taxa.

Currently, a number of water bodies are polluted by excessive organic matter, N and P, such as Tai Lake, where eutrophication has led to algal blooms that have adversely affected industrial and agricultural production since the 1990s, and the regional direct economic loss was estimated at approximately 30 million US dollars (Duan et al., 2009). Some previous studies have indicated that the imbalanced nutrients caused by eutrophication always have marked influences on bacterial communities and functions (Jarvie et al., 2018; Wang et al., 2018), which was also confirmed by the results of our study. Therefore, this study reveals a response mechanism of aquatic bacterial assemblages to imbalanced nutrients (*e.g.*, COD/N and N/P), as well as a basic methodology for wetland management (*e.g.*, constructed wetlands) involving organic and nutritional balance and bacterial manipulation (Zeng et al., 2021). This approach is also conducive to converting pollutants into resources and could support improved provisioning of ecological services by the freshwater of wetlands.

## Conclusion

The responses of bacterial assemblage structure to different COD/N and N/P ratios were revealed in this study. These results suggested that fluctuation of N sources was a predominant factor controlling the structure of bacterial assemblages in the freshwater environment. A high COD/N significantly increased bacterial alpha diversity, while this measure of diversity was negatively impacted by N/P. COD/N had a negative effect on beta diversity, and NH_4_^+^-N and EC greatly affected the distribution of bacterial assemblages. Organic to nutrient imbalance led to the differentiation of bacterial communities. However, LH increased the complexity of bacterial co-occurrence networks, and the more abundant connections of bacterial assemblages might be attributed to the balance of COD/N and N/P in the environment with increases in N sources. However, in aquatic environments, organic matter, N, and P are customarily regarded as pollutants. Constructed wetlands are used to improve the removal rate of pollutants by regulating organic to nutrient ratios. Our results shed light on the effects of organic to nutrient imbalance on bacterial diversity and interactions and represent considerable progress in advancing the functional management of bacterial communities and improving water quality, thereby enabling future research efforts to forecast the effects of freshwater bacterial communities on ecosystem function.

## Data availability statement

The datasets presented in this study can be found in online repositories. The names of the repository/repositories and accession number(s) can be found below: NCBI, PRJNA865028.

## Author contributions

FZ: methodology, data curation, investigation, formal analysis, writing—original draft, and resources. TZ, GQ, JC, and JZ: investigation. SY: visualization. DZ, XL and LX: writing—review and editing and validation. SA: project administration and funding acquisition. All authors have read and agreed to the published version of the manuscript.
