# Supplementary material for: Comparison and interpretation of freshwater bacterial structure and interactions with organic to nutrient imbalances in restored wetlands
Source: Front Microbiol. 2022 Sep 21;13:946537. doi: 10.3389/fmicb.2022.946537 (PMC9533089; doi:10.3389/fmicb.2022.946537)

Table S1 Correlation coefficients between the diversity indices and physicochemical parameters

|  | Sobs | Shannon | Chao1 | PD |
| --- | --- | --- | --- | --- |
| COD | 0.068 | 0.137 | 0.176 | 0.150 |
| TP | 0.352* | 0.146 | 0.501^**^ | 0.384^*^ |
| NH_4_^+^-N | -0.293 | -0.193 | -0.292 | -0.263 |
| NO_3_^-^-N | -0.205 | -0.185 | -0.217 | -0.235 |
| TN | -0.241 | -0.210 | -0.171 | -0.197 |
| COD/N | 0.276 | 0.275 | 0.312 | 0.323 |
| N/P | -0.342* | -0.194 | -0.358^*^ | -0.332 |

Note: ** represents a significant correlation at *p* < 0.01 and * represents a significant correlation at *p* < 0.05

Table S2 The statistical results of Adonis analysis

|  | Df | Sums Of Sqs | Mean Sqs | F. Model | R^2^ | Pr (>F) |
| --- | --- | --- | --- | --- | --- | --- |
| Group factor $ freshwater | 3 | 2.777 | 0.925 | 9.927 | 0.498 | 0.001 |
| Residuals | 30 | 2.798 | 0.093 |  | 0.501 |  |
| Total | 33 | 5.575 |  |  | 1 |  |

Note: SumOfSqs represents the total variance, MeanSqs represents the average variance, F_Model represents the F test value, and the R^2^ value represents the degree of explanation of each factor for the sample difference, and *p* < 0.001 indicates high reliability.

Table S3 The rank importance of bacterial OTUs and annotated species in freshwater (top 30).

| OTU_ID | Importance | Phylum | Class | Order | Family | Genus | Species |
| --- | --- | --- | --- | --- | --- | --- | --- |
| OTU20976 | 2.945 | Planctomycetes | Planctomycetacia | Gemmatales | Gemmataceae | norank_Gemmataceae | uncultured_bacterium_norank_Gemmataceae |
| OTU6768 | 2.937 | Cyanobacteria | Oxyphotobacteria | Phormidesmiales | Nodosilineaceae | Nodosilinea_PCC-7104 | uncultured_bacterium_Nodosilinea_PCC-7104 |
| OTU20946 | 2.936 | Actinobacteria | Actinobacteria | Microtrichales | Ilumatobacteraceae | CL500-29_marine_group | marine_metagenome_CL500-29_marine_group |
| OTU7901 | 2.849 | Bacteroidetes | Bacteroidia | Chitinophagales | Saprospiraceae | Candidatus_Aquirestis | uncultured_bacterium_Candidatus_Aquirestis |
| OTU15711 | 2.847 | Bacteroidetes | Bacteroidia | Flavobacteriales | Flavobacteriaceae | Flavobacterium | unclassified_Flavobacterium |
| OTU8009 | 2.809 | Proteobacteria | Gammaproteobacteria | Pseudomonadales | Moraxellaceae | Acinetobacter | unclassified_Acinetobacter |
| OTU14865 | 2.805 | Proteobacteria | Alphaproteobacteria | unclassified_Alphaproteobacteria | unclassified_Alphaproteobacteria | unclassified_Alphaproteobacteria | unclassified_Alphaproteobacteria |
| OTU7306 | 2.775 | Proteobacteria | Alphaproteobacteria | Rhizobiales | Hyphomicrobiaceae | Hyphomicrobium | metagenome_Hyphomicrobium |
| OTU6404 | 2.772 | Actinobacteria | Actinobacteria | Microtrichales | Ilumatobacteraceae | CL500-29_marine_group | unclassified_CL500-29_marine_group |
| OTU4919 | 2.692 | Proteobacteria | Alphaproteobacteria | Rickettsiales | Mitochondria | norank_Mitochondria | unclassified_norank_Mitochondria |
| OTU5727 | 2.625 | Proteobacteria | Alphaproteobacteria | Rhizobiales | Rhizobiales_Incertae_Sedis | Phreatobacter | Alphaproteobacteria_bacterium_Phreatobacter |
| OTU4904 | 2.615 | Proteobacteria | Gammaproteobacteria | Betaproteobacteriales | Methylophilaceae | norank_Methylophilaceae | uncultured_bacterium_norank_Methylophilaceae |
| OTU18015 | 2.546 | Bacteroidetes | Bacteroidia | Sphingobacteriales | NS11-12_marine_group | norank_NS11-12_marine_group | uncultured_Sphingobacterium_sp._norank_NS11-12_marine_group |
| OTU9228 | 2.538 | Bacteroidetes | Bacteroidia | Flavobacteriales | Crocinitomicaceae | Fluviicola | unclassified_Fluviicola |
| OTU14924 | 2.514 | Firmicutes | Bacilli | Bacillales | Paenibacillaceae | Paenibacillus | unclassified_Paenibacillus |
| OTU6323 | 2.510 | Chloroflexi | Anaerolineae | Caldilineales | Caldilineaceae | norank_Caldilineaceae | uncultured_bacterium_norank_Caldilineaceae |
| OTU20898 | 2.489 | Chloroflexi | Anaerolineae | Caldilineales | Caldilineaceae | norank_Caldilineaceae | metagenome_norank_Caldilineaceae |
| OTU9317 | 2.486 | Cyanobacteria | unclassified_Cyanobacteria | unclassified_Cyanobacteria | unclassified_Cyanobacteria | unclassified_Cyanobacteria | unclassified_Cyanobacteria |
| OTU7888 | 2.477 | Proteobacteria | Deltaproteobacteria | Oligoflexales | 0319-6G20 | norank_0319-6G20 | uncultured_bacterium_norank_0319-6G20 |
| OTU6440 | 2.449 | Chloroflexi | Anaerolineae | Caldilineales | Caldilineaceae | norank_Caldilineaceae | uncultured_bacterium_norank_Caldilineaceae |
| OTU6380 | 2.443 | Actinobacteria | Actinobacteria | Microtrichales | Ilumatobacteraceae | CL500-29_marine_group | uncultured_bacterium_CL500-29_marine_group |
| OTU9149 | 2.437 | Proteobacteria | unclassified_Proteobacteria | unclassified_Proteobacteria | unclassified_Proteobacteria | unclassified_Proteobacteria | unclassified_Proteobacteria |
| OTU6361 | 2.405 | Cyanobacteria | Oxyphotobacteria | Chloroplast | norank_Chloroplast | norank_norank_Chloroplast | Heterosigma_akashiwo_norank |
| OTU15722 | 2.404 | Actinobacteria | Actinobacteria | Frankiales | Sporichthyaceae | unclassified_Sporichthyaceae | unclassified_Sporichthyaceae |
| OTU2733 | 2.402 | Proteobacteria | Gammaproteobacteria | Betaproteobacteriales | Burkholderiaceae | unclassified_Burkholderiaceae | unclassified_Burkholderiaceae |
| OTU15727 | 2.366 | Planctomycetes | unclassified_Planctomycetes | unclassified_Planctomycetes | unclassified_Planctomycetes | unclassified_Planctomycetes | unclassified_Planctomycetes |
| OTU15698 | 2.360 | Proteobacteria | Deltaproteobacteria | Myxococcales | Archangiaceae | Anaeromyxobacter | uncultured_bacterium_Anaeromyxobacter |
| OTU18004 | 2.346 | Actinobacteria | Actinobacteria | Microtrichales | Ilumatobacteraceae | CL500-29_marine_group | uncultured_bacterium_CL500-29_marine_group |
| OTU20970 | 2.342 | Cyanobacteria | Melainabacteria | Vampirovibrionales | norank_Vampirovibrionales | norank_norank_Vampirovibrionales | uncultured_bacterium_norank_norank_Vampirovibrionales |
| OTU5094 | 2.340 | Proteobacteria | Alphaproteobacteria | Rhizobiales | Beijerinckiaceae | alphaI_cluster | unclassified_alphaI_cluster |

Fig. S1 a) Phylum and b) class-level bacterial communities in freshwater of restored wetlands. The horizontal distribution of phyla is based on the OTU clustering algorithm with 97% similarity. Differently colored areas in the bar graph are the proportions of different phyla and classes. Species with abundances less than 1% were merged into “Others”.
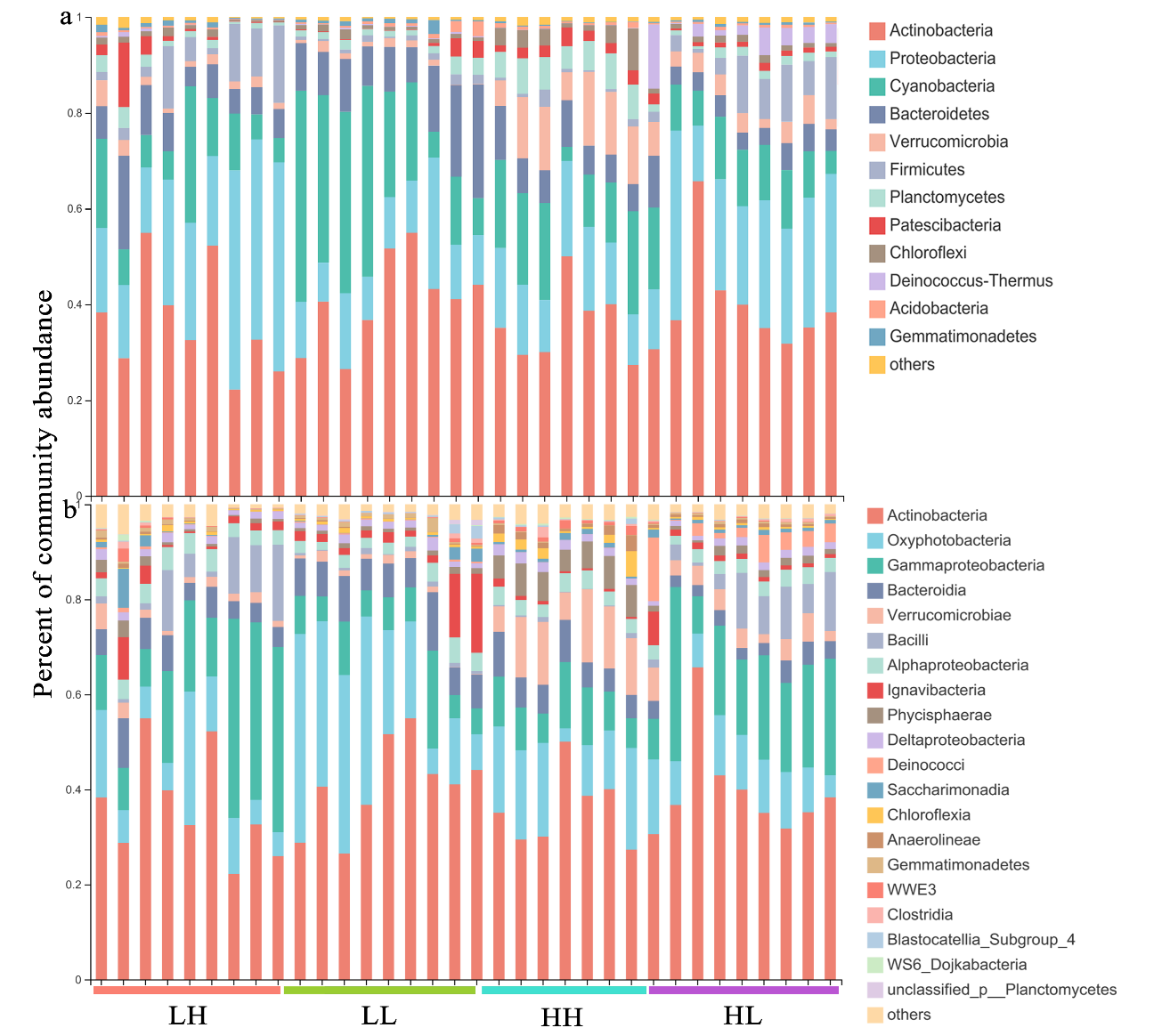

Supplement: Supplementary file 6 [file Data_Sheet_6.docx]
